# Health-related quality of life in young adults with symptoms of constipation continuing from childhood into adulthood

**DOI:** 10.1186/1477-7525-7-20

**Published:** 2009-03-02

**Authors:** Marloes EJ Bongers, Marc A Benninga, Heleen Maurice-Stam, Martha A Grootenhuis

**Affiliations:** 1 Department of Pediatric Gastroenterology & Nutrition, Emma Children's Hospital, Academic Medical Centre, Meibergdreef 9, 1105 AZ Amsterdam, the Netherlands; 2 Pediatric Psychosocial Department, Emma Children's Hospital, Academic Medical Centre, Meibergdreef 9,1105 AZ Amsterdam, the Netherlands

## Abstract

**Background:**

Children with functional constipation report impaired Health-related Quality of Life (HRQoL) in relation to physical complaints and long duration of symptoms. In about one third of children with constipation, symptoms continue into adulthood. Knowledge on HRQoL in adults with constipation persisting from childhood is lacking.

**Objectives:**

To assess HRQoL in adults with constipation from early childhood in comparison to that of their peers. Furthermore to gain insight into the specific social consequences related to continuing symptoms of constipation and/or fecal incontinence at adult age.

**Methods:**

One HRQoL questionnaire and one self-developed questionnaire focusing on specific consequences of symptoms of constipation continuing into adulthood were administrated to 182 adults with a history of childhood constipation. Successful clinical outcome was defined as a defecation frequency three or more times per week with less than two episodes of fecal incontinence per month, irrespective of laxative use. HRQoL of both adults with unsuccessful and successful clinical outcome were compared to a control group of 361 peers from the general Dutch population.

**Results:**

No differences in HRQoL were found between the whole study population and healthy peers, nor between adults with successful clinical outcome (n = 139) and the control group. Adults with an unsuccessful clinical outcome (n = 43) reported significantly lower HRQoL compared to the control group with respect to scores on bodily pain (mean ± SD 77.4 ± 19.6 versus 85.7 ± 19.5, p = 0.01) and general health (67.6 ± 18.8 versus 74.0 ± 18.1, p = 0.04). Adults with an unsuccessful clinical outcome reported difficulties with social contact and intimacy (20% and 12.5%, respectively), related to their current symptoms. Current therapy in these adults was more often self-administered treatment (e.g. diet modifications) (60.4%) than laxatives (20.9%).

**Conclusion:**

Overall, young adults with constipation in childhood report a good quality of life, as HRQoL of adults with successful clinical outcome was comparable to that of their peers. However, when childhood constipation continues into adulthood, it influences HRQoL negatively with social consequences in 20% of these adults.

## Background

Functional constipation in children is a common disease with a worldwide prevalence of 8.9% (range 0.7–29.6%) [1]. In about a third of these children symptoms continue into adulthood despite intensive treatment and follow-up [2,3]. Chronic symptoms of constipation, especially frequent episodes of fecal incontinence, are a source of great distress and concern to the child and its family. Besides physical distress, more behavioral problems are reported in children with constipation and fecal incontinence [4-6]. Children with functional constipation and their parents reported impaired quality of life in relation to physical complaints and long duration of symptoms [7,8]. Moreover, parent-reported quality of life in children with constipation was even lower than that reported by their children [7]. Only one small study observed a trend for adults with a history of childhood constipation to report lower levels of general health and social functioning when compared with controls [3].

To date, insufficient knowledge exists on the health-related quality of life (HRQoL) of adult patients experiencing constipation since childhood. Therefore, the aim of this study was to compare current HRQoL in young adults with a history of constipation with peers from the general Dutch population. Comparisons were made between young adults with continuing symptoms of childhood constipation, those free of symptoms of constipation, and healthy peers. Secondly, we aimed to gain more insight in the specific consequences of continuing symptoms of constipation and/or fecal incontinence at adult age.

## Methods

### Procedure

A cross-sectional study was performed at the Department of Pediatric Gastroenterology and Nutrition of the Emma Children's Hospital/Academic Medical Centre in Amsterdam. Patients were selected from an existing follow-up cohort of children with functional constipation formed between 1991–1999 [2]. Children were included in this follow-up cohort after participation in one of the research protocols on childhood constipation [9,10]. Diagnosis of functional constipation was based on presence of at least two of the following criteria: 1) defecation frequency less than three per week; 2) two or more episodes of fecal incontinence per week; 3) passage of very large amounts of stool once every 7 – 30 days; 4) a palpable abdominal or rectal mass on physical examination [9]. Patients under five years of age and/or patients with laxative treatment shorter than two months prior to inclusion in one of the research protocols were excluded, as those with organic causes of constipation. After ending the 6–8 weeks treatment protocols, follow-up was conducted at six months and annually thereafter during a visit to the outpatient clinic or telephonically using a standardized questionnaire.

Between 2004 and 2007, the patients in this cohort aged between 18 and 30 years were asked, during a standard follow-up, to participate in this study. The follow-up of the patients was not influenced by their participation this study, and was conducted in all contacted patients. Participating patients received two questionnaires by post. After completion at home, these questionnaires could be returned in a stamped addressed envelope provided. In case of no response, the patient was reminded telephonically with a maximum of two follow-up calls. Inclusion criteria for participation in the study were: 1) age 18–30 years before 1 January 2007; 2) the ability to read and understand the Dutch language of the questionnaires. Patients refusing to participate were asked to give their reason for declining study participation by phone. All participants signed an informed consent form. The study protocol was approved by the medical ethical committee of the Academic Medical Centre of Amsterdam.

### Measures

#### Quality of life

HRQoL was assessed with the RAND-36. The RAND-36 is a Dutch version of the MOS-SF-36 Health Survey and almost identical to the Dutch SF-36 [11]. The RAND-36 is composed of 36 items with standardized response choices, clustered into eight multi-item scales; Physical Functioning (PF), Social Functioning (SF), Role limitations due to Physical health problems (RP), Role limitations due to Emotional problems (RE), general Mental Health (MH), Vitality (VT), Bodily Pain (BP) and General Health perception (GH). The questions refer to the previous four weeks. All raw scale scores are converted to a 0–100 scale, with higher scores indicating a better HRQoL. Missing data on the RAND-36 were imputed at scale level. If less than half the items of a scale was missing, the scale-score was calculated based on items the respondent had completed.

A normative population for the RAND-36 was formed previously, including a sample of 508 young adults from the general Dutch population [12]. This control group consisted of otherwise healthy patients randomly chosen by 96 general practitioners. Young adults younger than 18 years or older than 30 years, or with a history of cancer or those who had not completed the questionnaire were excluded from this study [12]. As our study population was younger than this normative population, we selected those adults who were between 18 and 27 years of age as a control group for this study.

Validity and reliability of the RAND scales are satisfactory [13]. We found Cronbach's alphas in the range 0.76–0.87 in the study population and 0.73–0.90 in the control group.

#### Specification of consequences in relation to persistence of constipation

A questionnaire was developed to assess the consequences of constipation continuing into adulthood. Eight questions were based on clinical experience of two of the authors (MAB: pediatric gastroenterologist specialized in functional defecation disorders and MAG: psychologist specialized in psychosocial consequences of chronic diseases). Four questions focused on current complaints and treatment (Q1: What kind of defecation problems do you have currently?; Q2: If you still have symptoms, do you self-medicate?; Q3: If you still have symptoms, are you using medication presently? Q4: If you still have symptoms and are currently not using medical treatment; what are the reasons for not using medical therapy?). In addition, patients with unsuccessful clinical outcome were asked whether these complaints accounted for specific social consequences (Q5: How do you feel about talking to others about your problems of constipation and/or fecal incontinence?; Q6: How do you feel about talking to others about the treatment of your problems of constipation and/or fecal incontinence?; Q7: How often have you experienced social contact difficulties caused by problems of constipation and/or fecal incontinence?; Q7: How often have you experienced intimate contact difficulties caused by problems of constipation and/or fecal incontinence?; Q8: If your defecation problems have never caused difficulties with intimacy, what was the reason?).

#### Medical data

The following medical data were obtained from the follow-up database at the Department of Pediatric Gastroenterology & Nutrition: *intake characteristics: *age of onset, age, defecation and fecal incontinence frequency, painful defecation and abdominal pain; *last follow-up characteristics: *duration of follow-up, age, defecation and fecal incontinence frequency, painful defecation, abdominal pain and clinical outcome. Clinical outcome at last follow-up was regarded as successful if in the previous four weeks defecation frequency was three or more times per week with less than two episodes of fecal incontinence per month, irrespective of laxative use. According to this definition, the total group of adults who experienced constipation as a child was divided into two subgroups, i.e. one subgroup of patients with unsuccessful clinical outcome at adult age versus those with successful clinical outcome at adult age.

### Statistical Analysis

Descriptive analysis was performed to assess the characteristics of the sample. To detect *a priori *differences between adults with a history of childhood constipation and the control group, demographic characteristics were compared using Student's t-tests for continuous outcomes and Chi-square or Fisher's exact-tests for dichotomous outcomes. Furthermore, similar tests were used to assess for difference in intake characteristics during the first visit to the outpatient clinic and characteristics at last follow-up for adults with unsuccessful clinical outcome compared to those with successful clinical outcome.

Multivariate (MANOVA) and univariate analyses of variance (ANOVA) were conducted to test group differences on the RAND-36 scales, controlled for age at study and gender. Comparison was made between the total study population and the control group, but the control group was also compared to adults with unsuccessful clinical outcome, as well as adults with successful clinical outcome. Finally, adults with unsuccessful clinical outcome were compared to those with successful clinical outcome. A significant level of 0.05 was used. Effect sizes (d) were calculated by dividing the difference in mean score between groups concerned by the standard deviation of scores in the group allocated as reference. Effect sizes of 0.2, 0.5 and 0.8 were considered small, moderate and large, respectively [14].

Results on the short questionnaire with regards to specific consequences in adults with unsuccessful clinical outcome are given in a descriptive way.

## Results

From the existing follow-up cohort of 416 children with constipation, 299 patients reached the age of 18 years before January 2007. Of these adults, 68 patients (22.7%) dropped out from the follow-up cohort before 2004 for several reasons: wrongly included in previous research protocols: n = 9, protocol violation: n = 2, lost to follow-up: n = 56 and deceased: n = 1. No significant differences were found in age at intake, gender, age of onset, intake defecation and fecal incontinence frequency between drop-outs of the follow-up cohort compared to those available for follow up.

For this study, 231 young adults of our follow-up cohort were eligible. A total of 182 questionnaires were returned (response 78.8%). Of the 49 adults with childhood constipation not completing the questionnaires (non-responders), 19 (38.8%) did not have enough time or did not feel like participating in the study. A total of 30 adults (61.2%) agreed to fill out the questionnaires, but failed to return them. There was a higher percentage of women among the respondents compared to the non-responders (41.8% versus 22.4%, p = 0.01). Furthermore, at last follow-up, 23.6% of the responders had unsuccessful clinical outcome versus 10.2% of the non-responders (p = 0.04). Age of onset and age at intake, defecation and fecal incontinence frequency at intake, follow-up duration and age at last follow-up were not significantly different between responders and non-responders.

The demographic and medical characteristics of the study population and control group are given in tables 1 and 2. The total study population appeared to be different from the control group with respect to age at study and gender (table 1). Comparison within the study population showed that adults with unsuccessful clinical outcome were older at intake during the first visit to the outpatient clinic than those with successful clinical outcome (table 2). Furthermore, the percentage of males was significantly lower for adults with unsuccessful clinical outcome compared to those with successful clinical outcome. Clinical symptoms of constipation at last follow-up, i.e. defecation and fecal incontinence frequency, and accompanying symptoms such as painful defecation and abdominal pain, differed significantly between adults with unsuccessful clinical outcome compared to those with successful clinical outcome (table 2). Defecation frequency less than twice per week was present in 88% of adults with unsuccessful clinical outcome, while fecal incontinence once per two weeks or more often occurred in 21%.

**Table 1:** Demographic characteristics of the study population and the control group

	**Study Population (n = 182)**			**Control group (n = 361)**		
	
	**Mean**	**SD**	**Range**	**Mean**	**SD**	**Range**
Age at study (years)	21.4*	2.3	17.7–27.8	22.2	2.5	18.0–27.0
Age of onset (years)	3.3	2.8	0.0–12.0			
Age at intake (years)	9.2	2.4	5.1–17.1			
Follow-up duration (years)	12.1	1.8	7.0–15.0			

	**%**		**N**	**%**		**N**

Gender						
Male	58.2**		106	47.1		170
Female	41.8		76	52.9		191
Native Country						
The Netherlands	98.9		180	97.2		351
Other	1.1		2	2.8		10

*p = 0.001; **p = 0.02

**Table 2:** Demographic and medical characteristics of the study population according to clinical outcome at last follow-up

	**Unsuccessful (n = 43)**			**Successful (n = 139)**		
	
	**Mean**	**SD**	**Range**	**Mean**	**SD**	**Range**
**Intake characteristics**						
Age of onset (years)	3.9	3.7	0.0–12.0	3.1	2.4	0.0–10.0
Age at intake (years)	10.0*	2.6	5.1–17.1	9.0	2.3	5.1–16.1
Defecation/week	2.2	2.5	0.0–14.0	3.1	3.4	0.0–16.0
Fecal incontinence/week	13.3	12.5	0.0–37.0	13.0	10.8	0.0–56.0

	**%**		**N**	**%**		**N**

Gender (male)	44.2**		19	62.6		87
Large stools	74.4		32	61.2		85
Painful defecation	54.8		17	48.1		52
Abdominal pain	61.3		19	49.5		54
Abdominal scybalus	23.8		10	20.9		29
Rectal scybalus	34.9		15	26.6		37

	**Mean**	**SD**	**Range**	**Mean**	**SD**	**Range**

**Last follow-up characteristics**						
Age at last follow-up (years)	21.7	2.4	18.1–27.3	21.3	2.2	17.7–27.8
Follow-up duration (years)	11.7	1.9	7.0–15.0	12.3	1.8	8.0–15.0
Defecation/week	2.4***	1.5	0.3–7.0	6.5	3.3	3.0–28.0
Fecal incontinence/week	0.5***	1.6	0.0–7.0	0.0	0.0	0.0–0.3

	**%**		**N**	**%**		**N**

Painful defecation	46.5***		20	17.3		24
Abdominal pain	62.8****		27	33.1		46

*p = 0.02; **p = 0.03; ***p < 0.001; ****p = 0.001

### Quality of life (RAND-36)

The multivariate analysis of variance (MANOVA) for the RAND scales as a function of group, gender and age showed a main effect on gender (females scored lower than males), but not on group and age at study, for comparison between the total study population and the control group (F(8,522) = 4.1, p < 0.001).). In other words, no differences were found between the whole study population and healthy peers. A similar gender effect was also found for comparison between the successful clinical group and control group (F(8,479) = 3.0, p = 0.003). This was also found for the adults with unsuccessful clinical outcome compared to those with successful clinical outcome (F(8,166) = 4.1, p < 0.001). However no group differences between the successful clinical group and the control group or the unsuccessful clinical group were found.

Multivariate main effects on group (F(8,388) = 2.8, p = 0.005), gender (F(8,388) = 2.5, p = 0.01) and age at study ((F(8,388) = 2.0, p = 0.04) were found for comparison between adults with unsuccessful clinical outcome and the control group (table 3). Adults with unsuccessful clinical outcome showed worse HRQoL than the control group with respect to bodily pain (F(1,395) = 6.4, p = 0.01) and general health perception (F(1,395) = 4.5, p = 0.04). Effect sizes for these significant differences were 0.43 and 0.35, respectively.

**Table 3:** Mean scores, SD's and differences between adults with unsuccessful clinical outcome and the control group on the eight scales of the RAND-36

	**Unsuccessful^1^**			**Control group**			**Effect size (d)**
	
	**Males**	**Females**	**Total**	**Males**	**Females**	**Total**	**Total**
		
	**(n = 19)**	**(n = 24)**	**(n = 43)**	**(n = 169)**	**(n = 187)**	**(n = 356)**	
PF							
Mean	98.2	86.7	91.7	94.0	92.2	93.1	0.10
SD	4.2	16.8	14.0	13.6	14.7	14.0	
SF							
Mean	88.8	81.3	84.6	88.7	84.0	86.5	0.10
SD	21.2	24.5	23.1	18.2	20.5	19.3	
RP							
Mean	97.4	88.5	92.4	90.7	82.5	86.8	0.21
SD	7.9	22.1	17.7	21.6	31.7	27.0	
RE							
Mean	89.5	90.3	89.9	88.2	82.6	85.5	0.14
SD	27.3	25.0	25.8	28.9	32.8	30.8	
MH							
Mean	78.7	72.5	75.3	76.8	73.0	75.1	0.01
SD	16.7	15.1	15.9	15.6	16.5	15.9	
VT							
Mean	68.5	60.4	64.0	66.9	61.7	64.5	0.03
SD	18.3	16.6	17.7	16.6	18.3	17.4	
BP							
Mean	87.9	69.1	77.4*	89.0	82.9	85.7	0.43
SD	15.0	19.0	19.6	16.1	21.6	19.5	
GH							
Mean	74.7	61.9	67.6**	75.0	72.9	74.0	0.35
SD	17.6	18.1	18.8	17.0	18.9	18.1	

^1 ^Multivariate effects were found on group (p = 0.005), gender (p = 0.01) and age at study p = 0.04). *p = 0.01 and **p = 0.04: difference between adults with unsuccessful clinical outcome and the control group (based on univariate F-tests according to MANOVA by group, gender, age). PF: physical functioning; SF: social functioning; RP: role limitations due to physical problems; RE: role limitations due to emotional problems; MH: mental health; VT: vitality; BP: bodily pain; GH: general health perceptions.

### Specific consequences in adults with unsuccessful clinical outcome

In the 43 adults with childhood constipation continuing into adulthood, self-reported complaints were constipation in 76.7% and fecal incontinence with or without low defecation frequency in 14% (table 4). Four adults regarded themselves as free of symptoms, despite the fact that two of them had a low defecation frequency (two times per week) and the other two still experienced fecal incontinence with a frequency of two times per week and once per two weeks, respectively. The percentage of adults that administered self treatment, i.e. dietary measurements or toilet training, was high compared to the percentage using laxatives (60.6% versus 20.9%). Medical treatment was regarded as not necessary by 66.7% of the adults with unsuccessful clinical outcome. Twenty-five percent of adults found it difficult to talk about the persisting symptoms with others and 15% experiencing difficulties when talking about treatment of these symptoms. Problems with social contacts caused by constipation and/or fecal incontinence were reported by 20% of these adults, and 12.5% indicated to have had negative intimacy related experiences.

**Table 4:** Self-reported frequencies of specific consequences in adults with unsuccessful clinical outcome

	**Unsuccessful (n = 43)**	
	
	**%**	**N**
*1) Type of symptoms still present*		
constipation	76.7	33
fecal incontinence	7.0	3
both	7.0	3
none	9.3	4
*2) Self treatment for symptoms*		
diet	48.8	21
toilet training	11.6	5
none	39.5	17
*3) Treatment with laxatives for symptoms*		
yes	20.9	9
no	79.1	34
*4) Reason no medical treatment for symptoms*		
do not feel like it	9.1	3
do not know who or where to go to	15.2	5
not necessary	66.7	22
other reasons	9.1	3
*5) Feelings regarding talking to others about symptoms*		
(very) difficult	10.0	4
somewhat difficult	15.0	6
not at all difficult	35.0	14
do not talk about it	40.0	16
*6) Feelings regarding talking to others about treatment*		
(very) difficult	7.5	3
somewhat difficult	7.5	3
not at all difficult	25.0	10
do not talk about it	60.0	24
*6) Frequency of difficulties with social contact, related to symptoms*		
(very) often	15.0	6
Sometimes	5.0	2
never	80.0	32
*7) Frequency of difficulties with intimacy, related to symptoms*		
(very) often	2.5	1
sometimes	10.0	4
never	87.5	35
*8) Reason symptoms never a problem with intimacy*		
partners understand symptoms	47.1	16
hiding my symptoms	11.8	4
no fecal incontinence; thus no influence of symptoms on intimacy	26.5	9
never been intimate with someone	14.7	5

## Discussion

This study primarily assessed Health-related Quality of Life of young adults with a history of functional childhood constipation in comparison with the HRQOL of peers from the general Dutch population. Secondly, it aimed to gain more insight in the specific consequences of continuing symptoms of constipation and/or fecal incontinence at adult age. Symptoms continued into adulthood in 24% of children with constipation. No difference in HRQoL was found between the whole study population and healthy controls. While HRQoL was similar between young adults with successful clinical outcome and their peers, unsuccessful clinical outcome at adult age was associated with lower HRQoL with regards to general health and bodily pain compared to healthy controls. Furthermore, unsuccessful clinical outcome resulted in social consequences in one-fifth of adults with persistence of symptoms. Adults still experiencing symptoms of constipation and/or fecal incontinence applied more often self-administered treatments than laxatives.

Our data confirms previous findings in smaller cohort studies that childhood constipation continues into adulthood in approximately a quarter of patients [2,3]. Even after all these years, 88% of adults with unsuccessful clinical outcome experienced a low defecation frequency and fecal incontinence was still present in 21%. Surprisingly, fecal incontinence has not been addressed or recognized as significant symptom in young adults with constipation [15,16]. This is remarkable since it is well-know that in both children and adults, fecal incontinence negatively influences quality of life [17,18]. In addition, accompanying symptoms of constipation such as painful defecation and abdominal pain were approximately twice as common in adults with unsuccessful clinical outcome as in those free of constipation. Remarkably, only one out of five adults with unsuccessful clinical outcome still used laxatives and 66% found medical treatment no longer necessary.

These results may be explained in different ways. Those adults with a history of constipation going back to childhood may have adapted to the condition. Indeed, these adults reported no social consequences of their problems in the majority of cases. This is further underlined by the fact that we found no impairment of quality of life (QoL) on social, emotional or mental health scales in adults with persisting gastrointestinal symptoms, in contrast to several studies in patients with onset of functional gastrointestinal diseases at adult age [19-22]. However, comparison with some adult studies should be considered with caution since age and sex distributions were different to our study population [21,22]. Denial or shame of these symptoms still persists in adulthood. Disappointment in medical care may have contributed to the avoidance of medical care in these adults. To date, however, accurate knowledge of why these adults are no longer seeking medical treatment is lacking.

Adults with unsuccessful clinical outcome had poorer HRQoL, especially in general health perception and bodily pain, compared to healthy controls. A lower score for general health perception indicates that patients were more concerned about their health than were adults in the normal population. To date, only one study has reported on HRQoL in adults with a history of childhood constipation and found a trend of lower levels of general health and social functioning in these adults compared to controls [3]. However, comparison with our findings is hampered, as Khan et al. used a small sample of 20 adults without making a distinction between adults with continuing symptoms of constipation and those free of complaints [3]. A lower general health has also been reported in several studies in adults with functional constipation, as well as adults with (constipation predominant) irritable bowel syndrome. [23-26].

The lower score on bodily pain found in these adults with persisting symptoms of childhood constipation may be explained by the high frequency of pain complaints, i.e. painful defecation and abdominal pain. This finding seems in line with previous studies reporting impaired QoL in children with functional constipation and those with functional abdominal pain [7,27]. In children with chronic gastrointestinal disorders, low self-reported physical scores in response to questions regarding "ache or hurt", may reflect years of painful defecation and abdominal pain. Similarly, studies in adults with functional constipation or irritable bowel syndrome found that painful defecation and abdominal pain were strongly associated with impaired QoL [20,23,25,28].

QoL has been evaluated in other patient groups reporting defecation problems starting in early childhood and continuing into adulthood, i.e. Hirschsprung's disease and anorectal malformations [29]. Both of these patient groups, showed lower physical health, which was not found in our study population. In line with our findings, patients with anorectal malformations reported impaired QoL with respect to general health and pain level [29]. Remarkably, self-esteem and social support and not disease-specific factors like constipation and fecal incontinence, were the main mediating factors affecting generic QoL [29]. Yet, it is questionable whether you can compare HRQoL outcome of patients with congenital diseases to those with a functional gastrointestinal disorder.

It should be acknowledged that overall young adults with constipation in childhood report a good quality of life, as HRQoL of adults with successful clinical outcome was comparable to that of healthy controls. Furthermore, no overall significant difference in HRQoL scores was found between successfully and unsuccessfully treated adults. Due to the lack of a disease specific questionnaire, a generic questionnaire was used to compare the HRQoL between young adult with and without successful clinical outcome of their childhood constipation. However, a generic questionnaire may lack the sensitivity to assess important group differences within a specific patient population if these differences are not large [30]. In contrast to adults with persisting symptoms, scores on bodily pain and general health perception for successful clinically treated adults were comparable to healthy controls (Bodily pain: 86.8 versus 85.7; General health: 72.8 versus 73.9, respectively). This finding seems to support the idea that the impaired HRQoL found in adults with unsuccessful clinical outcome is related to the persistence of symptoms. Furthermore, the additional findings with the specific questionnaire further support the importance of using disease specific questionnaires in studying the impact of a chronic disease.

The long follow-up duration of the patient cohort and the fairly low drop-out rate are important strengths of this study. To our knowledge this is the first controlled study to assess the HRQoL in large cohort of adults with childhood constipation. Nonetheless, some limitations of the study need to be considered. Our findings could be biased by the patients lost to follow-up, as we do not know whether these drop-outs were more or less likely to have achieved successful clinical outcome. Furthermore, our findings are possibly biased by the fact that the percentage of adults with unsuccessful clinical outcome was higher among responders than among non-responders. However, this bias is most likely limited as the overall response rate of the study was high and the responders group had three times more successfully treated adults than those with persisting symptoms. Finally, no correction for other factors potentially influencing HRQoL was made. It has been suggested that psychosocial factors such as anxiety/depression, self-esteem and social support could affect patient-perceived health status [29,31,32]. Further analysis of patients' psychosocial functioning, whether or not related to constipation, in our study population may give more insight into the interaction between these health aspects.

## Conclusion

Functional constipation in children is not always a benign condition with favorable outcome, as symptoms persist into young adulthood in approximately a quarter of these children. Although, young adults with constipation in childhood report a good quality of life, persistence of childhood constipation into adulthood is associated with impaired HRQoL at adult age. Symptoms affect social contacts in a fifth of adults with unsuccessful clinical outcome. In our opinion, practitioners should give greater consideration to the impact of chronic constipation into young adulthood. Further research to quantify this burden is needed to determine the best course for prevention and treatment strategies.

## Abbreviations

HRQoL: Health-related Quality of Life; (M) ANOVA: (multivariate) analysis of variance.

## Competing interests

The authors declare that they have no competing interests.

## Authors' contributions

MEB collected the data for this study, conducted the analysis and interpretation of data and wrote the manuscript. MAB designed the study, collected the data for this study, and contributed to critical revision of the manuscript. HMA contributed to the analysis and interpretation of the data and critical revision of the manuscript. MAG contributed to the design of the study, the analysis and interpretation of the data and critical revision of the manuscript. All authors read and approved the final version of the manuscript.
